# New insights into *Eragrostis curvula*’s sexual and apomictic reproductive development

**DOI:** 10.3389/fpls.2025.1530855

**Published:** 2025-05-01

**Authors:** María Cielo Pasten, José Carballo, Alejandra Raquel Díaz, Chiara Mizzotti, Mara Cucinotta, Lucia Colombo, Viviana Carmen Echenique, Marta Adelina Mendes

**Affiliations:** ^1^ Centro de Recursos Naturales Renovables de la Zona Semiárida (CERZOS), Universidad Nacional del Sur - Consejo Nacional de Investigaciones Científicas y Técnicas (UNS - CONICET), Bahía Blanca, Argentina; ^2^ Departamento de Agronomía, Universidad Nacional del Sur, Bahía Blanca, Argentina; ^3^ Departamento de Biología, Bioquímica y Farmacia, Universidad Nacional del Sur, Bahía Blanca, Argentina; ^4^ Dipartimento di Bioscienze, Università Degli Studi di Milano, Milan, Italy

**Keywords:** *Eragrostis curvula*, confocal laser microscopy, ovule development, pollen development, apomixis, sexual reproduction

## Abstract

Apomixis, defined as asexual propagation by seeds, is considered of great importance for agriculture as it allows the fixation of desired traits and its propagation through generations. *Eragrostis curvula* (Schrad.) Ness, is a perennial grass that comprises a polymorphic complex including sexual and diplosporous apomictic cytotypes, where all apomicts are polyploids. In this study we present the first detailed description of female and male gametophyte development in *E. curvula* through confocal laser microscopy, contrasting three genotypes: the fully apomictic Tanganyika, the facultative apomictic Don Walter, and the sexual OTA-S. Moreover, we have studied the localized expression of a gene known as *SQUAMOSA PROMOTER BINDING PROTEIN-LIKE7* (SPL7), that was found to be differentially expressed in contrasting genotypes of E. curvula. This gene had been previously linked with flower development and abiotic stresses in several species, thus, in situ hybridizations were carried out in the model plant Arabidopsis thaliana, as well as in sexual and apomictic *E. curvula* genotypes. Our microscopy analysis has led to the identification of specific morphological characteristics for each genotype, mainly depicting a larger ovule in the sexual genotype’s reproductive development after the meiosis stage. These results reveal potentially important features, which could be used for a simple identification of genotypes. Moreover, differential expression of the gene SPL7 was detected, specifically determining an overexpression of the gene in the sexual genotype. These results demonstrated that it could be an interesting candidate to understand the mechanisms behind apomictic development.

## 1 Introduction

The morphology of the ovule of angiosperms was first described by microscopists Grew (1682) and Malpighi (1675), but it was not until 1827 that Brown provided the definite depiction of a divided ovule with distinct membranes, called integuments, that enclose a central nucellus (Brown, 1827). The area of origin of the integuments is called the chalaza or base of the ovule, while the opposite end is the micropyle, the entrance point for the pollen tube (Bouman, 1984). In the majority of angiosperms, the female reproductive organ consists of three parts: the ovary (where the ovule is located), the style (where the pollen tube grows) and the stigma on top (where the pollen grains adhere), which can have distinct features in different species (Gasser and Robinson-Beers, 1993). In plants, ovule development has been extensively investigated and it has been subdivided into two major steps: megasporogenesis, which involves the origination of functional spore(s); and megagametogenesis, entailing the formation of the mature embryo sac. The pattern of embryo sac formation varies in different species, and it has been classified into different types, the most frequent one being the *Polygonum*-type embryo sac, present in more than 70% of flowering plants, including *Eragrostis curvula*. This pattern has been extensively described, especially in the sexual model species *Arabidopsis thaliana* (Christensen et al.,1997; Drews and Koltunow, 2011; Vijayan et al., 2021). In 1997, Christensen et al. defined seven stages used for *polygonum*-type female gametophytes, named FG1 - 7. Briefly, at the FG0 stage an enlarged cell in the nucellus of the ovule primordia, called the megaspore mother cell (MMC), enters meiosis and leads to four megaspores. In the first stage (FG1) one of these megaspores, the Functional Megaspore (FM), starts to grow while the other three degenerate. From FG2 to FG4 this cell undergoes two rounds of mitosis, going from two to four nuclei separated by a central vacuole. After a third round of mitosis, stages FG5–7 initially consist of eight nuclei: three antipodals, two synergids, an egg cell, and two polar nuclei. The latter will fuse into a central cell. At the same time, in the anthers, pollen development takes place within the microsporangium, when the formation of reduced microspores by meiosis of a Pollen Mother Cell (PMC) is occurring. Microsporogenesis is followed by the development of mature pollen grains by separate mitosis, during microgametogenesis. Finally, the formation of two-cell pollen grain occurs followed by one round of mitosis. One of the two cells, the generative cell, divides originating the two sperm cells (Pacini and Dolferus, 2016; Halbritter et al., 2018). When fertilization occurs, the pollen tube, formed by the vegetative cell, enters the embryo sac delivering the two sperm cells. One male gamete fuses with the egg cell to form the diploid embryo, while the second sperm cell merges with the central cell and generates the triploid endosperm (Tucker and Koltunow, 2009; Mendes et al., 2016; Xu et al., 2022).

Numerous species can generate a female gametophyte by an asexual process known as apomixis, that gives rise to clonal seeds identical to the maternal plant. This type of reproduction has been observed in more than 400 species, most of them of the *Poaceae* and *Asteraceae* families, but the trait is absent in species of commercial importance such as wheat, maize, and rice. Known apomictic species include also some fruit crops such as citrus (Wakana and Uemoto, 1987), mango (Aron et al., 1998) and crabapple (Liu et al., 2014). Apomixis comprises three main parts: apomeiosis (absence of meiosis), parthenogenesis (absence of fertilization), and autonomous endosperm development or pseudogamy, all of which gives rise to an unreduced embryo sac that will successfully develop into a mature seed (Savidan, 1992; Carman, 1997; Xu et al., 2022). It has been observed that apomixis has arisen independently more than once during evolution, and there are multiple known mechanisms that take place in the diverse species (Marshall and Brown, 1981). According to the origin of the unreduced embryo sac and the moment of initiation of the process, this type of reproduction can be divided into two big categories: sporophytic apomixis (initiated by a sporophytic somatic cell) and gametophytic apomixis (originated by cells initially developing as a megagametophyte), this last one being subdivided once more in diplospory and apospory. In diplospory, the unreduced embryo sac develops from the MMC avoiding meiosis, while apospory is initiated by a cell of the nucellus adjacent to the MMC (Asker and Jerling, 1992; Susmita et al., 2022). The elucidation of apomictic mechanisms has been the aim of several studies throughout the years and numerous advances have been made, including increasing evidence that suggests that the regulation of this trait is influenced by epigenetic pathways, regulation of protein degradation, signal transduction and hormonal control, among others (Schmidt, 2020; Selva et al., 2020; Carballo et al., 2021; Yin et al., 2022; Carballo et al., 2019; Zappacosta et al., 2019). Nonetheless, even though it was possible to obtain mutants and clonal seeds with synthetic apomixis in rice (Khanday et al., 2019; Wang, 2020; Vernet et al., 2022), the natural apomictic traits were never completely transferred into agronomically important species. Furthering the comprehension of the underlying processes that influence this type of reproduction would allow for the trait to be transferred to crops of economic importance, such as wheat and maize, which is expected to have an immense impact in agriculture. Potentially, this process would reduce breeding costs, heterozygosity, fix desired traits from generation to generation and avoid incompatibility barriers (Barcaccia and Albertini, 2013).

*Eragrostis curvula* is a perennial pasture from the Poeaceae family valued in several parts of the world as a forage grass, particularly in semi-arid regions of Australia, Argentina and USA, due to its drought tolerance and soil conservation properties (Voigt and Bashaw, 1976; Bashaw and Hanna, 1990; Covas, 1991). The inflorescence consists of a panicle with a length that ranges from 15 to 40 cm, formed of spikelets with 3–6 flowers inside. Each flower comprises one to three anthers, and a pistil with two styles and two feathered stigmas (Lazarides, 1997; Voigt et al., 2004). This type of stigma is called plumose, and it is frequent in the Poaceae family. *E. curvula* includes both sexual genotypes and some with diplosporous apomixis, which can be either obligate or facultative, meaning that some embryo sacs are sexual while others have an apomictic development (Voigt and Burson, 1983). In diplospory, the MMC either avoids meiosis altogether or the process starts and is later blocked or modified (Cornaro et al., 2023). In the *Eragrostis*-*type* embryo sac development, that is specific to this genus, the MMC enters two rounds of mitosis to generate a tetranucleate embryo sac. The unreduced egg cell then undergoes parthenogenesis, which has been found both in non-vascular plants and in angiosperms, as well as in some animals. This process is usually linked to the presence of apomeiosis, but the inheritance of both processes has been proven to be independent (Vijverberg et al., 2019). Subsequently, the endosperm of the mature seed is formed by the fertilization of the unreduced central cell with a reduced sperm cell, and the embryo: endosperm ploidy ratio remains 2:3, as in sexual development (Carballo et al., 2021). *E. curvula* has been broadly studied with respect to its type of reproduction, becoming an important model of natural apomixis.

Using laser confocal microscopy and a Feulgen-based staining protocol (Braselton et al., 1996) we have studied the gametophytic development in *E. curvula* sexual, apomictic and facultative genotypes.

Also, taking into consideration differentially expressed genes found between contrasting genotypes (Garbus et al., 2017), we decided to study in more detail the gene *SQUAMOSA PROMOTER BINDING PROTEIN-LIKE7* (*SPL7*), which is one of the few genes encoding for a transcription factor differentially expressed in the two genotypes. This gene has been associated with copper homeostasis in plants (Yamasaki et al., 2009), a process crucial for plant growth (Schulten et al., 2022), seed production, and fertility in both pollen (Yan et al., 2017) and the gynoecium (Rahmati Ishka and Vatamaniuk, 2020). It has also been implicated in responses to abiotic stresses (Stief et al., 2014; Perea-Garcia et al., 2021; Wang et al., 2021). By *in situ* hybridization we are able to show that *SPL7* is specifically expressed only in ovules of sexual *E. curvula* genotypes, suggesting it could be one of the factors involved in the switch between sexual and diplosporic embryo sac formation.

## 2 Materials and methods

### 2.1 Plant material

The plants used in this work were three tetraploid (2n = 4x = 40) *E. curvula* genotypes: the sexual OTA-S (USDA: PI574506), the full apomictic Tanganyika (USDA: PI234217) and the facultative apomictic cv. Don Walter. Moreover, a diploid sexual accession, USDA PI208214, was used as a contrasting genotype for the *in situ* hybridization assays. They were grown in a greenhouse in 10-liter pots, under natural light conditions (Bahía Blanca, Argentina, 38°43’0″S, 62°16’0″O), at 25˚C +/- 4°C. Inflorescences were taken at the beginning of anthesis, when all embryo sac developmental stages are found simultaneously in an individual panicle (Selva et al., 2017).

### 2.2 Confocal microscopy

#### 2.2.1 Fixation and staining

The samples were stained implementing a protocol based on Barrell and Grossniklaus (2013) and modified by Cornaro et al. (2024). After leaving the spikelets in fixative FAA (50% ethanol, 5% acetic acid, 10% formaldehyde) for 24 hours at room temperature (RT), 95% ethanol was added, and they were stored in 70% ethanol at 4°C. Afterwards, the samples were rinsed in distilled water three times and treated with 1M HCl for 1.5 hours at RT. Then, the HCl 1M was replaced with HCl 5.8M for 2 hours, to be later placed a second time back in HCl 1M for an hour at RT. The spikelets were rinsed with distilled water three times once again and dyed with Schiff’s reagent (Merck) at 4°C, leaving them overnight. Using an ethanol series of 30, 50, 70, 95 and 100% ethanol (30 minutes for each step), the samples were dehydrated, and they were finally added to a solution consisting of 50% immersion oil (Merck) and 50% ethanol, and stored overnight at 4°C.

#### 2.2.2 Microscopy

Stained spikelets at all developmental stages were selected and separated from the rachis. They were then cautiously dissected under a Leica MZ9.5 stereo microscope using thin hypodermic needles and slim tweezers, to separate the glumes of the flower in order to release the pistil and anthers as a whole, as shown in **Supplementary Figure 1**. Then, 100 µl of 50% immersion oil and ethanol solution was then added to whole mounted slides, consisting of approximately 15–20 pistils for each genotype. After that, they were covered with glass coverslips.

Observations were carried out with a confocal laser Nikon Eclipse Ti2 inverted microscope, equipped with a Nikon A1R and a laser scanning device. The slides were placed on the 40X objective with a drop of distilled water, and the laser used for the examinations was RFP. Images were obtained with a CFI Apo Lambda 40×C LWD WI, and the platform NIS-Elements (Nikon) was used to control the microscope settings and its functions. The obtained images were analyzed with the NIS-Elements platform and subsequently measured using the software Fiji (ImageJ) (Schindelin et al., 2012).

#### 2.2.3 Characterization and measurements

**Figure 1:**
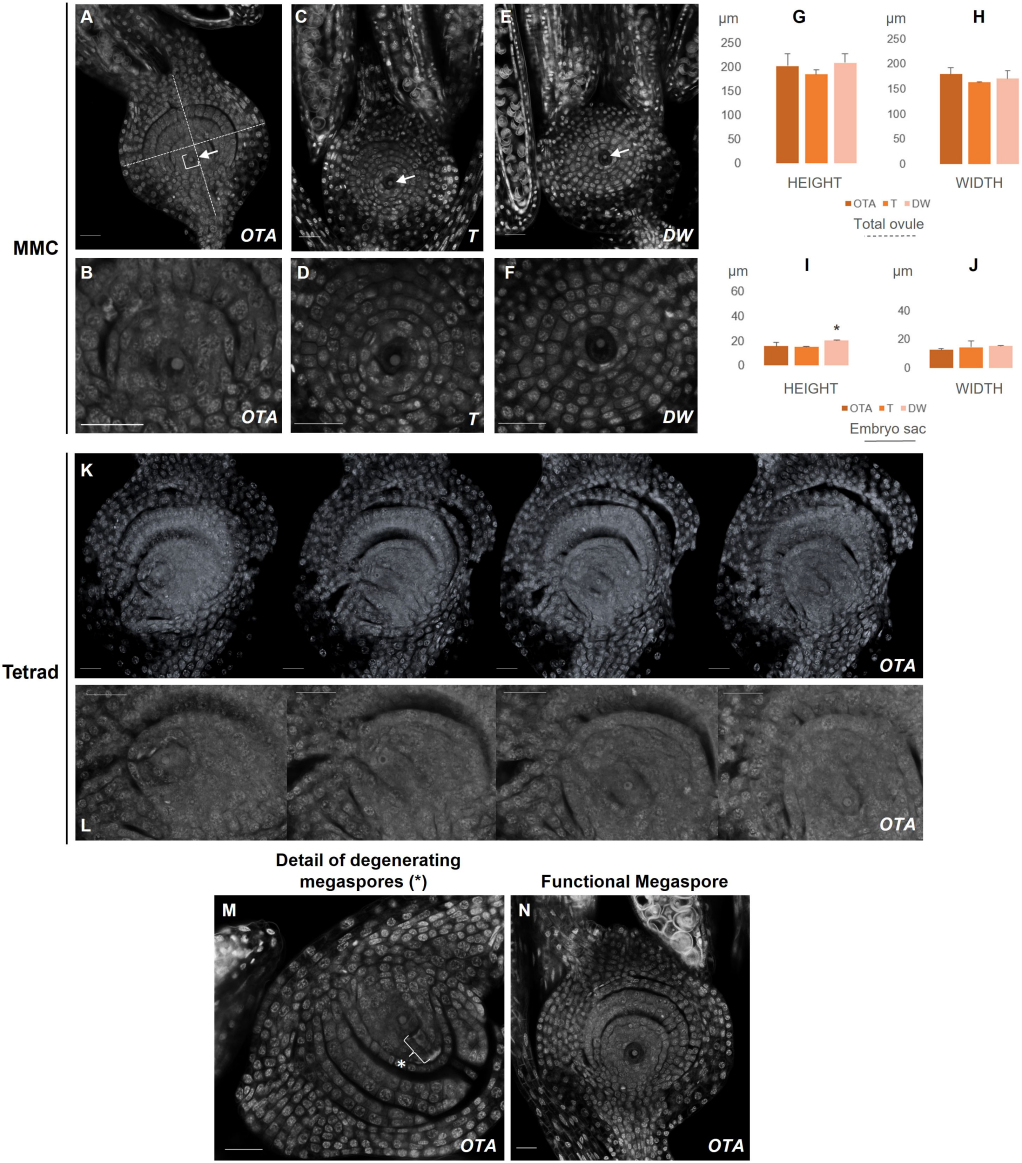
Confocal laser images of ovules of OTA, Tanganyika (T) and Don Walter (DW). Scale bars of 25µm. **(A, B)** MMC stage in OTA; **(C, D)** MMC stage in Tanganyika; **(E, F)** MMC stage in Don Walter. The nuclei of the MMC is indicated with an arrow in **(A, C, E)**. **(K)** Tetrad stage in OTA; **(L)** Closer images of the four nuclei of Tetrad stage in OTA. **(M)** Degenerating megaspores in OTA(*); **(N)** Functional Megaspore stage in OTA. The bar graphs indicate the comparative lengths of the MMC stage in micrometers (µm). **(G, H)** Measurements of the total ovule for each genotype; **(I, J)** Measurements of the embryo sac for each genotype. Statistical differences between genotypes are shown with a *p<0,05.

**Figure 2:**
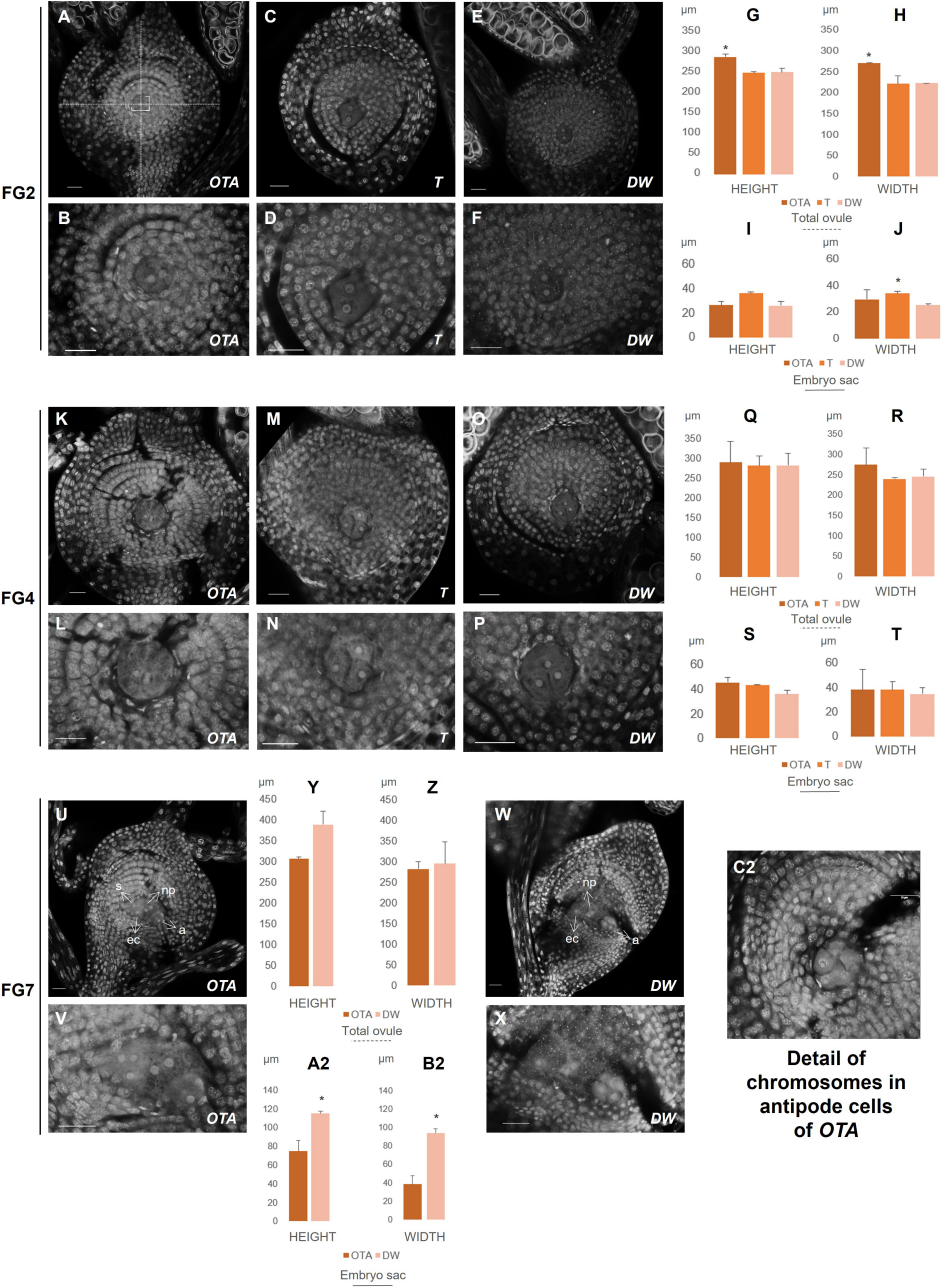
Confocal laser images of ovules of OTA, Tanganyika (T) and Don Walter (DW). Scale bars of 25µm. **(A, B)** FG2 stage in OTA; **(C, D)** FG2 stage in Tanganyika; **(E, F)** FG2 stage in Don Walter; **(K, L)** FG4 stage in OTA; **(M, N)** FG4 stage in Tanganyika; **(O, P)** FG4 stage in Don Walter; **(U, V)** FG7 stage in OTA; **(W, X)** FG7 stage in Don Walter; **(C2)** Detail of chromosomes in antipodal cells of sexual genotype OTA. The bar graphs indicate the comparative lengths of the different stages in micrometers (µm). **(G, H)** Measurements of the total ovule in FG2 stage; **(I, J)** Measurements of the embryo sac in FG2 stage; **(Q, R)** Measurements of the total ovule in FG4 stage; **(S, T)** Measurements of the embryo sac in FG4 stage; **(Y, Z)** Measurements of the total ovule in FG7 stage; **(A2, B2)** Measurements of the embryo sac in FG7 stage. Statistical differences between genotypes are shown with a *p<0,05.

For the three genotypes, the key developmental stages were analyzed and characterized, ranging from megaspore mother cell (MMC) to mature ovule pre-fertilization stages (FG7). The stages of development considered are shown in **Table 1**, and the entirety of the observations were performed for two different ovules for each stage. For each genotype, a timeline of these phases was established to determine the most significant features of each developmental step (**Figures 1**, **2**).

**Table 1:** Stages of development analyzed by confocal laser microscopy and measured, for each genotype of *E. curvula*.

Tanganyika (Apo)	OTA (Sex)	Don Walter (Fac)
MMC	MMC	MMC
–	Tetrad	–
–	Functional Megaspore (FM)	–
FG2	FG2	FG2
FG4	FG4	FG4
–	FG7	FG7

Additionally, in order to identify differences between types of reproduction, the width and height of the ovule and embryo sac were measured for the stages comparable between genotypes, *i.e*. MMC, FG2 stage and FG4 stage (**Supplementary Table 1**; **Figures 1**, **2**). A total of 24 ovules were used for the measurements, 10 of which corresponded to the sexual genotype stages, and 14 to the full and facultative apomicts. Every assessment was performed with the straight-line measure feature available in the Fiji software, which calculates the length in micrometers of each measured segment (**Figures 1A**, **2A**).

The ovules were found to be divided in four layers in every developmental phase, separated by a gap in the tridimensional space, thus, as well as measuring the total length and width of the ovule and the embryo sac, five cells of each layer were measured to obtain an average length of each of the four sections. The layers measured are exhibited in **Supplementary Figure 2**.

The use of the confocal laser technique also allowed the obtention of stacks or z-stacks, meaning sample slices that are obtained consecutively by the laser beam, showing in a detailed manner the structure and spatial distribution of the nuclei and layers in each phase. For example, the tetrad observed in the sexual genotype OTA-S could not be obtained as a single picture, but as a z-stack (**Figure 1K**). With these stacks, using the modeling feature of the software Fiji (ImageJ), tridimensional representations of the ovules can be generated if the number of sample slices is sufficient. We were able to construct a 3D model for each of the analyzed genotypes (**Supplementary Figure 2D**).

This feature could help discern patterns of development and could also be of use in the study of the organogenesis of the species.

#### 2.2.4 Statistical analysis

For all the different stages compared, using two different ovules for each, the assessments corresponded to the average of the five measurements for each of the four layers, the length and width of the whole ovule, and the length and width of the embryo sacs of each genotype. For the four stages that could be compared between genotypes (**Table 1**), a Student’s T-test was performed to contrast the obtained values, and *p*-values of 0.05 and less were considered to be statistically significant. All the results obtained are exhibited in **Supplementary Table 1**.

### 2.3 Regulation of apomictic development in *Eragrostis curvula*

In order to strengthen the study of the species, a RT-PCR and *in situ* hybridization were performed with the gene *SPL7* (*SQUAMOSA PROMOTER BINDING PROTEIN-LIKE7*) (**Supplementary Table 2**), found to be differentially expressed in the full-apomictic and full-sexual genotypes of *E. curvula*.

#### 2.3.1 Selection of the gene and phylogenetic tree

RNA libraries were constructed and sequenced as reported in Garbus et al. (2017) and were deposited in the Sequence Reads Archive (SRA) database at NCBI under the BioProject. These reads were mapped against the *E. curvula* genome assembly (Bioproject PRJNA508722) using the STAR software (Dobin et al., 2013) and a differential expression analysis was carried out between these libraries using the R package DESeq2 (Love et al., 2014). Genes were considered differentially expressed when they had a log2foldchange value of +/-1 and a p-value < 0.05. Finally, genes were annotated using the STRING platform (Szklarczyk et al., 2015) with *Arabidopsis Thaliana* as reference.

Based on previous work and the expression data, the gene annotated as *SQUAMOSA PROMOTER-BINDING PROTEIN-LIKE7* (*SPL7*) was selected to be analyzed, since it was also found differentially regulated and previous studies of this gene suggest a relation with reproductive development and pollen fertility (Yan et al., 2017; Tian et al., 2018; Gou et al., 2019), as well as different abiotic stresses (Perea-García et al., 2021). Moreover, all proteins containing the Pfam domain SBP (for Squamosa promoter Binding Protein ID: PF03110) in the genomes of *E. curvula, A. thaliana* and *O. sativa* were extracted from the Ensembl database (Martin et al., 2023, http://plants.ensembl.org) (**Supplementary Table 3**). These proteins were aligned by the Muscle software (Edgar, 2004). Using the pairwise alignment of SPLs proteins as input, a maximum likelihood phylogenetic tree (bootstrap = 1000) was constructed using the software MEGA (Tamura et al., 2021). Finally, Evolview was used to plot the phylogenetic tree (Subramanian et al., 2019).

**Figure 3:**
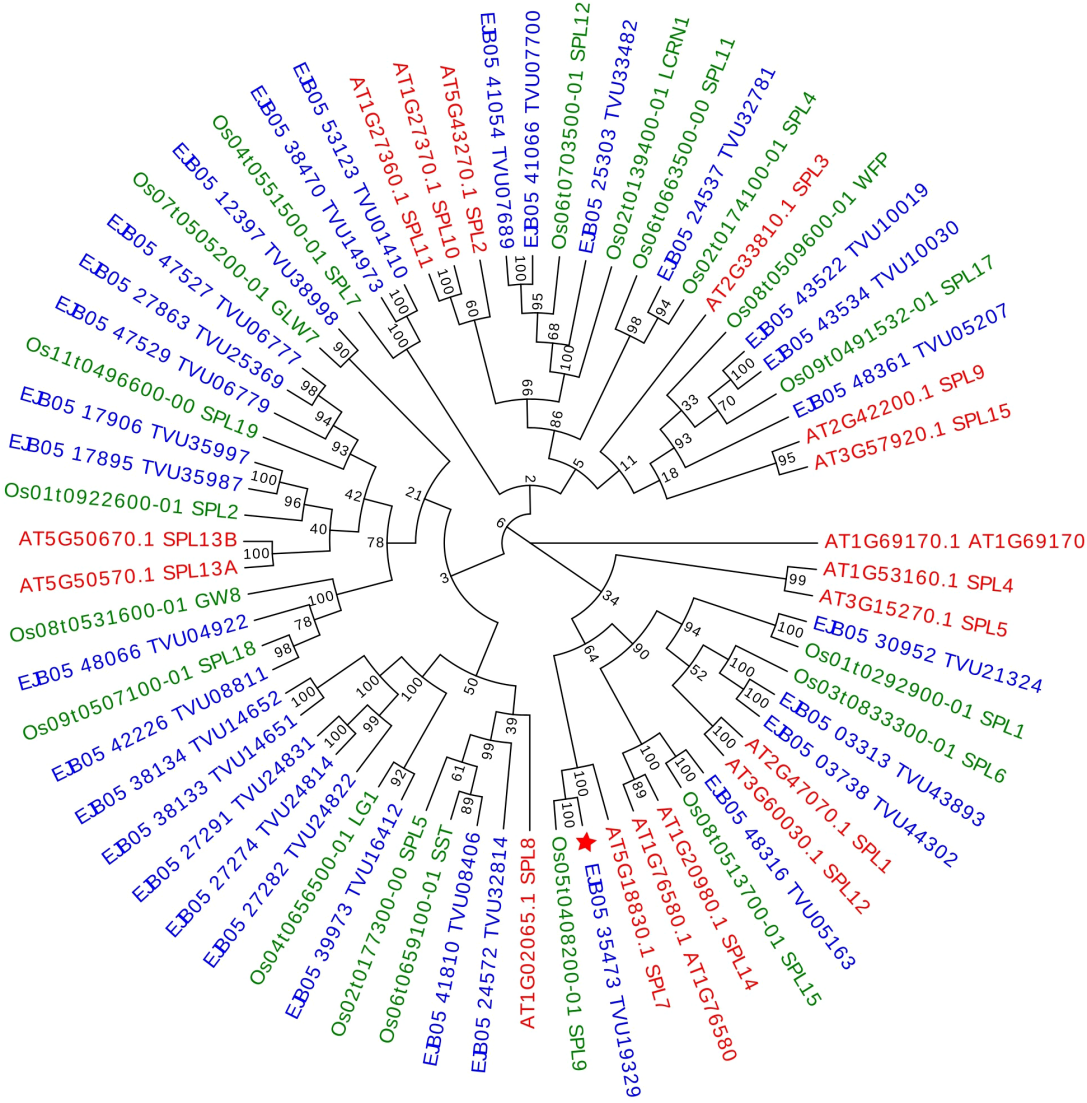
Maximum likelihood phylogenetic tree (bootstrap = 1000) of the SPL proteins constructed with *Eragrostis curvula* (shown in blue), *Oryza sativa* (shown in green) and *Arabidopsis thaliana* SPLs (shown in red). Bootstrap support values greater than 50 are shown at nodes. The transcript found to be differentially expressed in *E. curvula* is indicated with a red star (★).

The analysis allowed confirmation that the studied transcript of *E. curvula* is an ortholog of *A. thaliana*’s SPL7 and *O. sativa*’s SPL9, corresponding to the same protein (**Figure 3**). This difference in gene nomenclature was already stated in previous works (Peng et al., 2019; Zhu et al., 2020; Li et al., 2022).

#### 2.3.2 RNA extraction and cDNA synthesis

RNA was extracted in biological duplicates from the genotypes OTA-S and Tanganyika, in order to validate the *in silico* observations. Spikelets with basal flowers at the beginning of anthesis were collected, to include all developmental stages, and they were analyzed under a stereomicroscope (LeicaS APO). The extraction protocol was based on guanidinium thiocyanate and was performed as follows: Approximately 30–40 mg of tissue from biological duplicates were frozen in liquid nitrogen and homogenized using a plastic pestle in a 1.5 ml tube. Samples were then mixed with 500μl of TRIzol™ Reagent (Life Technologies). 100μl of chloroform was added, and then the RNA was precipitated with 250μl of isopropanol and washed with 500μl of 75% ethanol. The samples were resuspended in 20 μl of DEPC water and stored at −80°C. Quantification of the RNA was done using a DeNovix DS-11 spectrophotometer (Denovix Inc., USA), whereas agarose gel electrophoresis was used to examine its integrity and quality. For the synthesis of cDNA, an ImProm II™ Reverse Transcription System (Promega) was employed, following the instructions of the suppliers, using Random Primers (Promega).

#### 2.3.3 Semiquantitative RT-PCR

Specific primers were designed (**Supplementary Table 2**), based on the sequence of the differentially expressed transcript of *SPL7* found among libraries. For this purpose, the Integrated DNA Technologies (IDT) platform was used (https://www.idtdna.com/PrimerQuest/Home/Index).

PCR reactions were performed using cDNA as template. A MyCycler Thermal Cycler (Bio-Rad) was used, and the reactions were carried out with 0.3 μl of 40 mM dNTPs mix, 3 μl of 10× reaction buffer, 0.4 μl of forward and reverse primers (10 pmol/μl), 0.1 μl of DNA goTaq polymerase (Promega, 5U/μl), 2 μl of template cDNA (diluted 1/20), in a final volume of 15 μl. The cycling protocol for amplification comprised an initial denaturation step at 95°C for 2–5 min, followed by 30 cycles at 95°C for 30 s, 30 s at an optimal annealing temperature for the primer pair, and 72°C for 40s. The final step consisted of a final extension of 3 min at 72°C.

The primer annealing temperature was optimized starting from one degree below the lower melting temperature between both primers (60°C). Amplification products were analyzed by electrophoresis in a 1.5% (m/v) agarose gel and visualized using ECO-Gel Red dye (InBio Highway) in an UVIDOC HD6 gel imaging system.

#### 2.3.4 *In situ* hybridization

For the hybridization procedure, floral tissue of the two available contrasting genotypes, a fully sexual diploid (2n = 2x = 20) (USDA: PI208214) and a fully apomictic tetraploid (2n = 4x = 40) (Tanganyika USDA: PI234217), was used. Inflorescences were collected and fixed in formaldehyde-acetic acid-ethanol (FAA: 3.7% formaldehyde, 5% glacial acetic acid, 50% ethanol), and the embedding and sectioning were performed as described in Zumajo-Cardona et al. (2019).

The template for the synthesis of the specific RNA probe was obtained by amplification of a 300 bp fragment using specific primers containing the T7 sequence. They were then purified using the QIAquick PCR purification Kit (Qiagen). The DIG (digoxigenin) labeling was conducted using T7 RNA polymerase (Roche), RNAse inhibitor RNasin (New England Biolabs), and RNA labeling-mix (Roche) according to the manufacturer’s protocol. Finally, the hybridization and washes were performed according to Ambrose et al. (2000). The observations of the obtained slides were performed using a ZEISS Axioscope microscope equipped with an AxioCam MRm camera (Zeiss).

## 3 Results

### 3.1 Morphological characterization of ovule development

We performed a comprehensive confocal laser microscopy analysis and comparison of all the developmental stages of the female gametophyte in three genotypes of *Eragrostis curvula:* i) the sexual OTA-S, ii) the fully apomictic Tanganyika, and iii) the facultative apomictic Don Walter. As mentioned in the introduction, sexual development comprises the stages of FG0 or MMC (Megaspore Mother Cell), FG1 or FM (Tetrad and Functional Megaspore), FG2 (Two nuclei stage), FG4 (Four nuclei stage) and FG7 (Mature stage). We have used a protocol described by Barrell and Grossniklaus (2013) and modified by Cornaro et al. (2024), which allowed us to distinguish the nuclei (DNA) and the cell walls, and in some cases also the single chromosomes (**Figures 2C2**, **4A**). We focused our analysis on the megasporogenesis and megagametogenesis of the three genotypes, to identify morphological differences. The measurements were organized into three main calculations: the total ovule dimension (height and width) (exemplified in **Figures 1A**, **2A**), the total embryo sac dimension (height and width) (exemplified in **Figures 1A**, **2A**). Moreover, the organization of the ovule for the three genotypes was found to be divided in four distinguishable layers in the nucellus of every developmental phase (**Supplementary Figure 2A**), thus, five cells of each layer were measured in the same manner (height and width), to obtain an average dimension of each of the four sections (**Supplementary Figure 2A**). Comparisons were made between the obtained measurements in micrometers, performing a Student’s T-test (**Supplementary Table 1**).

**Figure 4:**
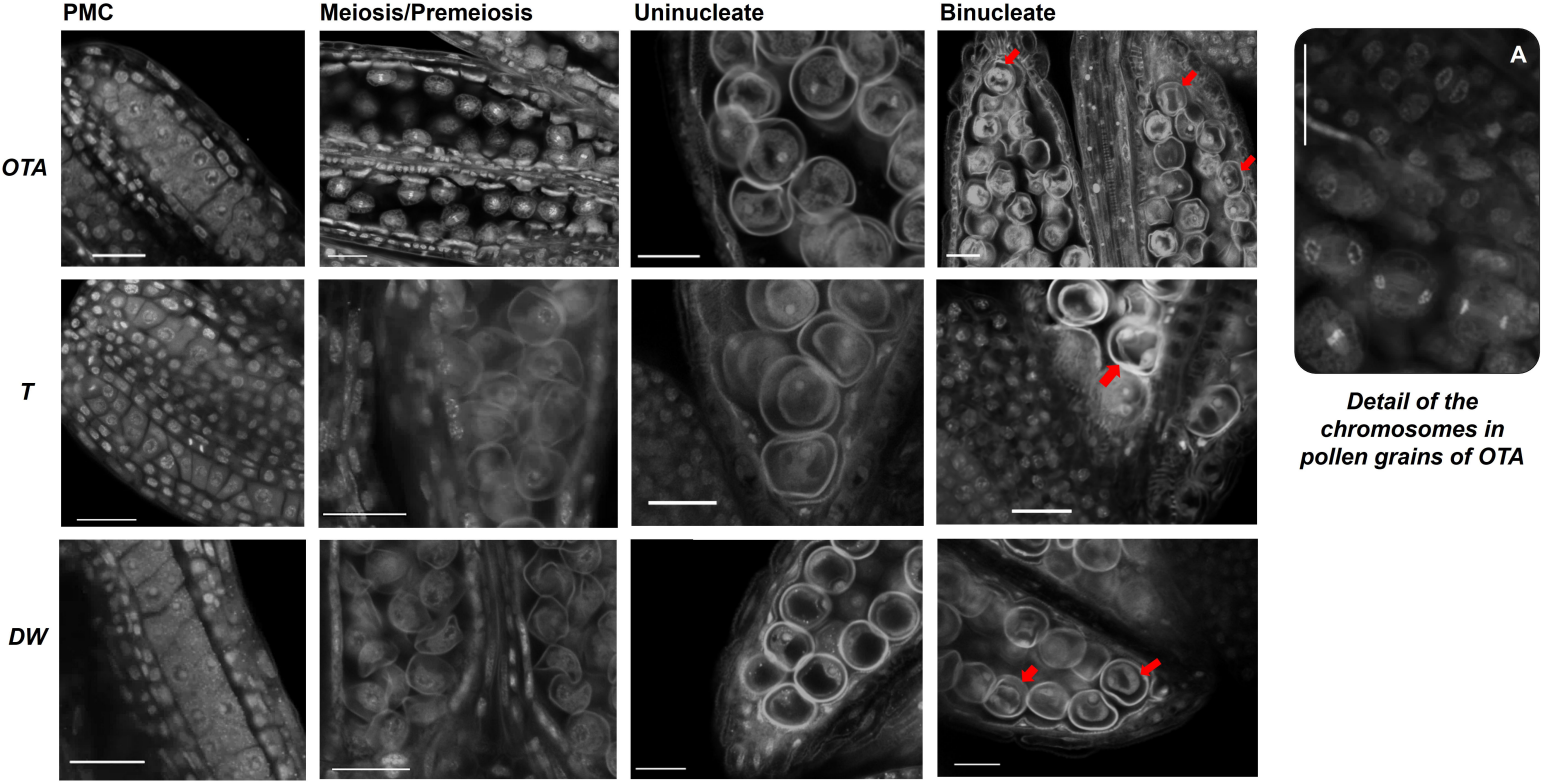
Confocal laser images of pollen grains of OTA, Tanganyika (T) and Don Walter (DW) for the following stages of development: Pollen mother cell (PMC); Meiosis/Pre-meiosis; Uninucleate and Binucleate pollen. Nuclei of Binucleate stages are indicated with a red arrow (➔). Scale bars of 25µm. **(A)** Detail of the chromosomes in pollen grains of genotype OTA.

The ovule is enclosed by the integuments and the development of the embryo sac occurs from the central portion of the ovule. During the initial steps of female development, ovules at pre-meiosis, for all three genotypes it was possible to observe a unique cell in the center of the embryo sac (**Figures 1A–F**), as well as two short immature stigmas attached to the ovule (**Supplementary Figure 2C**, MMC).

Regarding the height and width of the total ovule, it was observed that at pre-meiotic stage there were no notable differences in the measurements performed between the three analyzed genotypes (**Figures 1G–J**), where the MMC was clearly distinguished as a bigger nucleus (**Figures 1B, D, F**).

The average ovule height in the sexual genotype OTA was approximately 200µm, similarly to what was found in Don Walter while in Tanganyika was closer to 185µm. The average width was around 180µm in OTA, while it was closer to 165 and 170µm for Tanganyika and Don Walter respectively (**Supplementary Table 1**). On the other hand, for the embryo sac dimensions, Don Walter’s height (~20 µm) was found to be significantly larger than the fully apomictic Tanganyika (~ 15µm). OTA’s embryo sac height (~ 15.6µm) was closer to Tanganyika’s (**Supplementary Table 1**).

At this point in the sexual genotype OTA the MMC enters meiosis and forms a reduced tetrad, which was clearly distinguishable – the position of the nuclei of the four megaspores was found to be vertically aligned within the embryo sac (**Figures 1K, L**). The ovule measurements in the tetrad stage in OTA were a height of 282.5µm and a width of 214µm. After the tetrad formation in sexual development, three of the megaspores degenerate and one persists (the one closer to the chalaza), giving rise to the functional megaspore (FM) (**Figures 1L, M**). In FG1 a single nucleus was observed once again, often with a clear degeneration of the megaspores adjacent to it (**Figure 1L**), and it could be distinguished from the MMC stage because of its larger size (average height: MMC ~201µm *vs* FM ~223µm; average width MMC ~179µm *vs* FM 224µm) and larger stigmas with the distinctive feathers (**Supplementary Figure 1C**). On the other hand, in the apomictic Tanganika and the facultative Don Walter, the FG1 stage is completely absent, and the ovule enters mitosis directly into FG2. Indeed, the MMC was clearly observed, but a tetrad was never detected in these two genotypes. Concerning the cells in each nucellus layer, there were no significant differences found between genotypes in MMC and FG1 stages (**Supplementary Table 1**).

At FG2, as the ovule continues growing after the first round of mitosis, the total ovule dimensions obtained revealed that the ovule of the sexual genotype OTA was significantly larger than its apomictic parallels (**Figure 2G**, **Supplementary Table 1**), reaching 288µm of height (**Supplementary Table 1**). With respect to the width, OTA was significantly wider than Don Walter (**Figure 2H**), measuring approximately 270µm. Tanganyika’s width was similar to that of Don Walter’s, being both around 220µm.

Interestingly though, in this stage Tanganyika’s embryo sac width (30 µm) was observed to be significantly bigger than the facultative genotype (**Figure 2J**), with a size comparable to OTA’s embryo sac (22 and 26µm respectively). Concerning the layer cells in the stage of FG2, for the first and second layers, sexual OTA’s cells were found to be longer. Likewise, for the second and third layers OTA’s cells appear to be wider (**Supplementary Table 1**, **Supplementary Figure 2B**).

After the second mitosis, at FG4 stage, the four nuclei in all genotypes were observed close together forming a group in the center of the ovule as shown in **Figures 2K–P**, where the images show three clearly distinguishable nuclei, whereas the fourth one was in all cases in a deeper level tridimensionally.

The ovule and embryo sac sizes were not found to be differential between genotypes for this stage (**Figures 2Q–T**). In OTA the total ovule height was approximately 289µm, while for Tanganyika and Don Walter it was close to 281µm. The total width was ~273µm in OTA, ~238µm in Tanganyika and ~244 µm in the facultative Don Walter (**Supplementary Table 1**). With respect to the embryo sac, OTA and Tanganyika’s dimensions were more similar between them, while Don Walter showed a tendency to a smaller, rounder embryo sac (DW height ~35 µm and width ~34µm) (**Supplementary Table 1**).

At this stage, regarding the layer cell analysis, sexual genotype OTA’s cells were observed to be wider for the first, second and third layers, while also being longer for the second and third layers. Nonetheless it was observed that, interestingly, the full apomictic genotype Tanganyika at FG4 showed much longer cells forming the fourth layer (**Supplementary Table 1**, **Supplementary Figure 2B**).

Finally, the FG7 octonucleate stage could be detected in the sexual OTA and also in the facultative apomict Don Walter, where we were able to find a sexually developing embryo sac. As it was mentioned in the introduction, in the *Eragrostis*-*type* apomictic development, the MMC follows only two rounds of mitosis, generating a tetranucleate embryo sac, so the fully apomictic genotype Tanganyika did not exhibit an FG7 stage. Even though no significant differences were found for the dimensions of the total ovule (**Figures 2Y, Z**), the size of the facultative apomict embryo sac was found to be significantly larger than the sexual genotype (**Figures 2A2, B2**), with a height of ~114µm and a width of ~93µm (**Supplementary Table 1**). Surprisingly however, regarding the layer cells, the first, second and third layers were comprised of significantly longer cells in the sexual OTA, while they were wider for the third and fourth layers (**Supplementary Table 1**, **Supplementary Figure 2B**).

**Figure 5:**
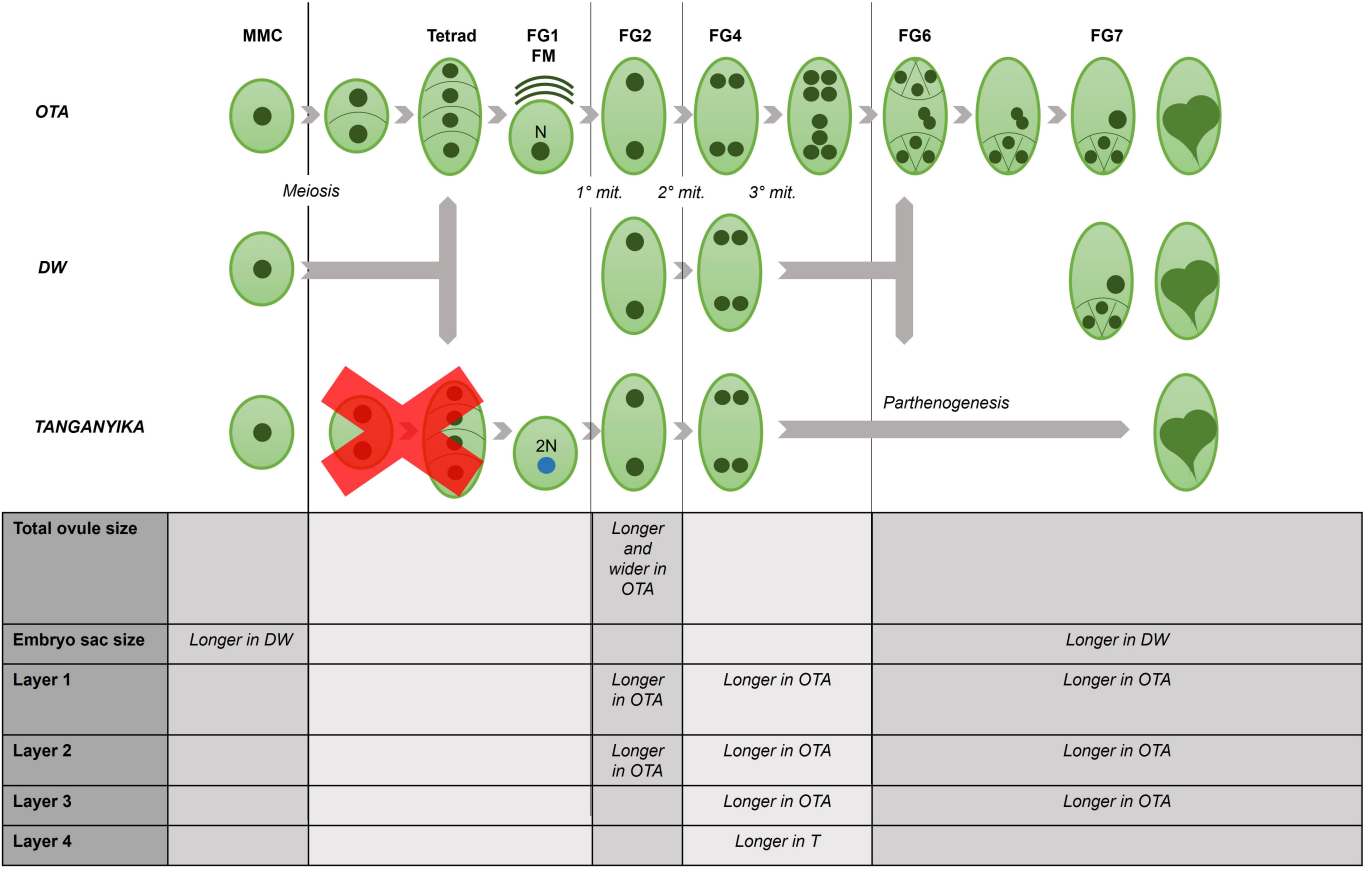
Schematic summary of the differential features observed under confocal microscopy, for each developmental stage in the ovule of the three studied genotypes of *E. curvula*.

The identified differential features found to be significant between genotypes are compiled in a schematic summary, exhibited in **Figure 5**.

### 3.2 Morphological characterization of pollen development

Regarding pollen grain development, microsporogenesis and microgametogenesis were analyzed for the three genotypes in study (**Figure 4**). The stages observed included the pollen mother cells, meiosis or pre-meiosis, the uninucleate grains and the bicellular pollen. The technique used in the work allowed the obtention of high-quality confocal images of the pollen development and spatial positioning, for the first time in *Eragrostis curvula*, and it even permitted the observation of chromosomes during the division stage. Nevertheless, it was not found particularly useful for the analysis of the surface texture of the pollen grains. Height and width of pollen grains were analyzed in the four stages observed, but no major differences were found between genotypes.

It was possible to recognize active division taking place in the early stages of pollen development in the sexual genotype OTA, observing metaphase and anaphase with remarkable clarity (**Figure 4A**).

### 3.3 SPL7, a putative apomixis regulator

#### 3.3.1 Phylogenetic tree

The *E. curvula* cDNA libraries previously published by Garbus et al. (2017) and Pasten et al. (2022), were inquired in order to select differentially expressed genes that could be potentially related with the mode of reproduction. This analysis allowed us to obtain a gene encoding for the putative ortholog of the *Squamosa Promoter binding protein-Like7* (*SPL7*, **Supplementary Table 2**). This gene was indeed found to be differentially expressed *in silico* in reproductive tissue of *E. curvula*, being overexpressed in the sexual OTA with respect to the fully apomictic Tanganyika.

Taking into consideration that *SPL7* was one of the few transcription factors found to be differentially expressed in the contrasting genotypes, and that it had been previously correlated to inflorescence development and flowering regulation, among other processes (Gou et al., 2019; Schulten et al., 2022), we decided to study this gene in more detail and analyze its expression pattern.

To analyze the phylogenetic relation among *Eragrostis curvula*’s *SPL7* and the ones identified other species a maximum likelihood tree was constructed. Using *Oryza sativa* ssp. Japonica, *Arabidopsis thaliana* and *Eragrostis curvula*, the comparative sequence analysis for the tree conclusively determined a correct clustering of the sequences corresponding to the gene *Squamosa promoter Binding-like 7* in the three species, as can be observed on **Figure 3**. The analysis allowed us to confirm that the *E. curvula SPL7* studied in this work (**Figure 3**, marked with a star) is unambiguously the ortholog of *A. thaliana* SPL7. Even more, *E. curvula*, and *A. thaliana* SPL7 genes group correspondingly with *O. sativa*’s SPL9. The incongruence in gene nomenclature was already reported in previous articles (Peng et al., 2019; Zhu et al., 2020; Li et al., 2022).

#### 3.3.2 Validation and *in situ* hybridization

**Figure 6:**
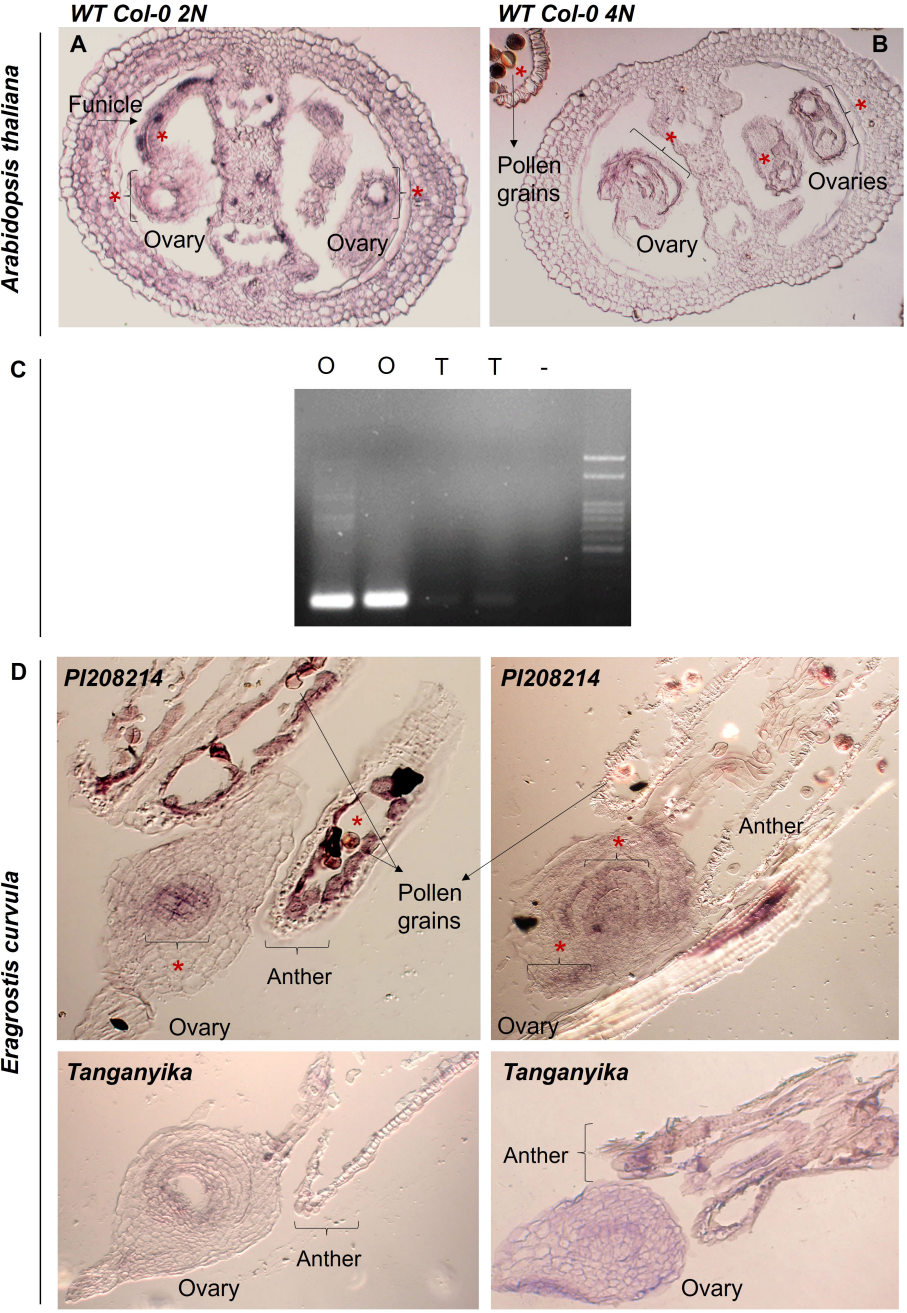
**(A, B)** Images of the *in situ* hybridizations performed in *A. thaliana*, for both diploid and tetraploid backgrounds of Wild Type Col-0. (*) Detected color signal. **(C)** Agarose gel (1,5% m/v) obtained for the amplification of the RT-PCR of sexual (O1 and O2) and apomictic (T1 and T2) cDNA duplicates with the gene *SPL7*. (**D**) Images of the *in situ* hybridizations performed *E. curvula* ovules of the sexual diploid accession PI208214 and the apomictic genotype Tanganyika, (*) Detected color signal.

As shown in **Figure 6C**, the amplification performed confirmed the previous data, as *SPL7* resulted highly overexpressed in the sexual genotype.

To deepen the study, *in situ* hybridizations were performed. For all cases, floral material was collected and fixed in FAA (formaldehyde-acetic acid-ethanol), after which they were embedded in paraffin and sectioned using a protocol based on Zumajo-Cardona et al., 2019. Then, a specific RNA probe of approximately 300bp was designed for *SPL7* (**Supplementary Table 2**) and DIG-labeled, and the tissue was later hybridized and washed using a protocol based in Ambrose et al. (2000).

Firstly, we performed an *in situ* hybridization in reproductive tissue of the model plant *Arabidopsis thaliana* ecotype Col-0, for both diploid (2N) and tetraploid (4N) backgrounds (**Figures 6A, B**; **Supplementary Figure 3**). The gene was found to be expressed in mature stages of development, for both diploid and tetraploid backgrounds, mainly surrounding the embryo sac and the integuments, as well as in the funicle and pollen grains.

Furthermore, an *in situ* hybridization was performed using inflorescences of two contrasting genotypes available of *Eragrostis curvula* (apomictic Tanganyika and sexual diploid accession PI208214) (**Figure 6**, **Supplementary Figure 3**). The results obtained from these experiments confirmed that *SPL7* was expressed exclusively in the ovules of the sexual genotype (**Figure 6D**). *SPL7* was detected at the center of the embryo sac in early stages of development, and it appears to expand to more regions all over the external part of the ovary in more advanced stages. On the contrary, there was no signal detected in either stage of development in the apomictic genotype Tanganyika (**Figure 6D**).

## 4 Discussion and conclusion

### 4.1 Insight into *E. curvula*’s reproductive development using confocal laser microscopy

Over the years, the elucidation of the genetic and epigenetic processes controlling ovule development in naturally apomictic species has been considered a key goal, as it would aid enormously in the development of valuable tools for the transference of this trait to economically important species, *i.e* maize, wheat, and rice (Hanna and Bashaw, 1987). Since asexual reproduction through seeds appears to have arisen more than once during evolution (Carman, 1997), the use of *Eragrostis curvula* as an experimental apomictic model adds great value to the study, as the complex consists of naturally sexual and apomictic genotypes, even including different ploidy levels. Moreover, this species preserves the normal relationship between the maternal and paternal genome in the endosperm, which has been found to be an important advantage for the transference to major crops and cereals (Carballo et al., 2021). Cyto-embryological studies of apomictic ovules of *E. curvula* have been performed with optical microscopy, using paraffin-embedded and sectioned material, typically stained with safranin-fast green or using clarification methods to observe pistils under DIC microscopy (Meier et al., 2011; Carballo et al., 2021). During several years, most of these protocols of microscopy imaging have been associated with low resolution and insufficient detail (Hepler and Gunning, 1998), but the introduction of confocal laser scanning microscopy allowed to effortlessly section thick stained samples, enabling the visualization of cells in its tridimensional orientation and providing context of the adjacent tissue, which is a huge advantage in relation to serial slicing (Running et al., 1995).

The characterization and description of ovule and pollen development has been extensively explored on different model species over the years, with the purpose of understanding the mechanisms lying underneath (Schneitz et al., 1995; Kuhn and de Araujo Mariath, 2014; Rossig et al., 2021; Rocha et al., 2023).

The optimized staining technique for whole-mount tissue on which this work was based, proved to be effective in the determination of the stages of megasporogenesis and megagametogenesis also in *A. thaliana* (Siddiqi et al., 2000; Vieira et al., 2012), as well as in maize (Barrell and Grossniklaus, 2005) and rice (Zeng et al, 2007), allowing the observation of distinct nuclei and cell walls, and overall granting high-quality images with a much less arduous protocol that excludes the time-consuming embedding and sectioning steps. Moreover, measurements using confocal laser microscopy techniques were successfully obtained for different species, such as embryo sac dimensions in rice (Zeng et al., 2007); *A. thaliana*’s ovule area, cell size and pistil length (Fernandez et al., 2010; Cucinotta et al., 2016; Gomez et al., 2023) and intercellular distances between sperm nuclei and female nuclei in *Theobroma cacao* (Ford and Wilkinson, 2012), among others. Likewise, in this work it was possible to observe and measure *E. curvula*’s reproductive tissue in depth, examining all planes of focus and spatial distribution of the structures.

A reproductive calendar of sexual and apomictic individuals of this species was previously presented by Selva et al. (2017) using fixed tissue, but no comparisons were performed between them.

Moreover, most of the reproductive calendars that have been constructed for apomictic grasses correspond to aposporous apomicts (Caceres et al., 1999; Dusi, 2001; Laspina et al., 2008; Sharma et al., 2014; Soliman et al., 2019), where there appears to be a different synchronization between male and female development in the apomictic individuals, which we did not detect during our analysis.

In the developmental description of the grass *Brachiaria decumbens* (Dusi, 2001), it was found that the aposporous genotypes had a higher growth rate, and that its pistils were larger during anthesis (FG7), even though the comparisons were made between tetraploid and diploid accessions, so this difference could be attributed to the ploidy level. In contrast, in our analyses it was observed that the sexual genotype OTA had a bigger total ovule size and larger dimensions corresponding to the first three layers in study for all stages. On the other hand, the embryo sac dimensions appeared to be larger for the two apomictic *E. curvula* genotypes for all stages analyzed. Interestingly, regarding the last layer that was observed in the ovule structures (**Supplementary Figure 1**), the full apomictic genotype Tanganyika exhibited much longer cells, reaching around 13µm (**Supplementary Table 1**, **Supplementary Figure 1**), constituting a potentially important feature for a simple identification of this genotype.

Moreover, it was possible to generate a developmental timeline for the pollen grain development of this species, effectively discerning four key stages in the three contrasting genotypes. Although at this time it was not possible to distinguish differences in shape, size or pollen wall architecture in the different genotypes, this procedure permitted the clear observation of nuclei and chromosomes, even enabling the examination of active division.

### 4.2 Regulation of apomictic development in *E. curvula*

In order to strengthen the knowledge about the regulation of the trait apomixis in this species, a transcription factor named *SQUAMOSA PROMOTER BINDING PROTEIN-LIKE7* (*SPL7*) was evaluated *in silico* and found to be overexpressed in the sexual genotype OTA. To validate this result, an RT-PCR was performed, and the experiment confirmed amplification solely in this genotype, suggesting a role or effect in the regulation of the trait. The assembly of a phylogenetic tree aiming to understand the identity and evolution interactions between the analyzed *E. curvula* transcript and the sequences corresponding to *SPL7* in *A. thaliana* and *SPL9 O. sativa* allowed to correctly cluster these genes as having ortholog identity. Subsequently, an *in situ* hybridization was performed to observe the localization of the expression of the gene, and it revealed a positive signal of expression only on the ovules corresponding to the sexual genotype, particularly in the center of the embryo sac.

The family of *SQUAMOSA PROMOTER BINDING PROTEIN-LIKE* transcription factors has been linked to reproduction in *Boechera* (Amiteye et al., 2011) and in a study of *Eragrostis curvula’s* differentially expressed microRNAs, where some of them were targeting transcripts encoding a SPL protein and a MADS-box transcription factor (Garbus et al., 2019). Particularly, the expression of *SPL7* in the grass *Panicum virgatum* (along with *SPL8*) was found to be related to inflorescence and stem development, as well as flowering time, and it was observed it acts targeting two transcription factors of the MADS family (Gou et al., 2019). This gene appears to be linked to miR156, and it has also been associated to abiotic stresses in *Arabidopsis thaliana* and *Carya illinoinensis* (Stief et al., 2014; Perea-Garcia et al., 2021; Wang et al., 2021), which could be of interest having in mind that a link has been stablished between drought stress and an increase in sexual embryo sacs in facultative apomictic genotypes of *E. curvula* (Rodrigo et al., 2017; Selva et al., 2020). Interestingly, *SPL7* has also been studied as a crucial copper regulator in plants (Yamasaki et al., 2009), and copper homeostasis is suggested to be of extreme importance in plant growth (Schulten et al., 2022), seed production, fertility of pollen (Yan et al., 2017) and also fertility of the gynoecium (Rahmati Ishka and Vatamaniuk, 2020), all of this being closely related to this gene.

Although a direct connection between this gene and apomictic or sexual reproduction has not been confirmed, it can be theorized, in light of the results obtained in this work, that *SPL7* is part of a regulatory pathway in sexual development, that could include microRNAs and genes of the family of the MADS-box, and which is repressed or not present in apomictic individuals. Moreover, in *A, thaliana*, *SPL7* was found differentially expressed in anthers and stamen at flower stage 15 (Mergner et al., 2020), meaning that regulation of apomixis through this gene could be triggered by the pollen, such as in other important genes like BBM1 (Khanday et al., 2019).

## Data Availability

The datasets presented in this study can be found in online repositories. The names of the repository/repositories and accession number(s) can be found in the article/**Supplementary Material**.

## Supplementary material

Supplementary Figure 1 Images obtained under the stereomicroscope during preparation of the samples. **(A)** a rachis containing four stained spikelets; **(B)** a closed stained spikelet, and **(C)** an open spikelet, displaying the stained pistil with a feathered stigmas at the center and three anthers on the sides, which was later observed under the confocal laser microscope.

Supplementary Figure 2
**(A)** References for the measurements of the dimensions of total ovule (TO, dotted lines) and embryo sac (ES, brackets). Four layers analyzed for all stages (parenthesis, 1-4); **(B)** Bar graphs indicating the comparative measurements of the layer cells (Layer 1-4) between the three contrasting genotypes (OTA, T, DW) for all stages (a, b, c, d): a: MMC; b: FG2, c: FG4, d: FG7. T-test statistical differences between genotypes is shown with a *: p<0.05. **(C)** Example of the difference in development and size observed in stigmas (indicated with *) of the genotype OTA for the MMC stage (MMC indicated with an arrow), and the FM stage. **(D)** Constructed 3D confocal models of tetrad stage in sexual OTA (left), FG2 stage in apomictic Tanganyika (middle), and FG4 stage in facultative Don Walter (right).

Supplementary Figure 3 Images of the *in situ* hybridizations with the sense probe performed in *A. thaliana*, for both diploid and tetraploid backgrounds, and *E. curvula* sexual diploid genotype.
