# Supplementary figures and images for: New insights into Eragrostis curvula’s sexual and apomictic reproductive development
Source: Front Plant Sci. 2025 May 1;16:1530855. doi: 10.3389/fpls.2025.1530855 (PMC12078246; doi:10.3389/fpls.2025.1530855)

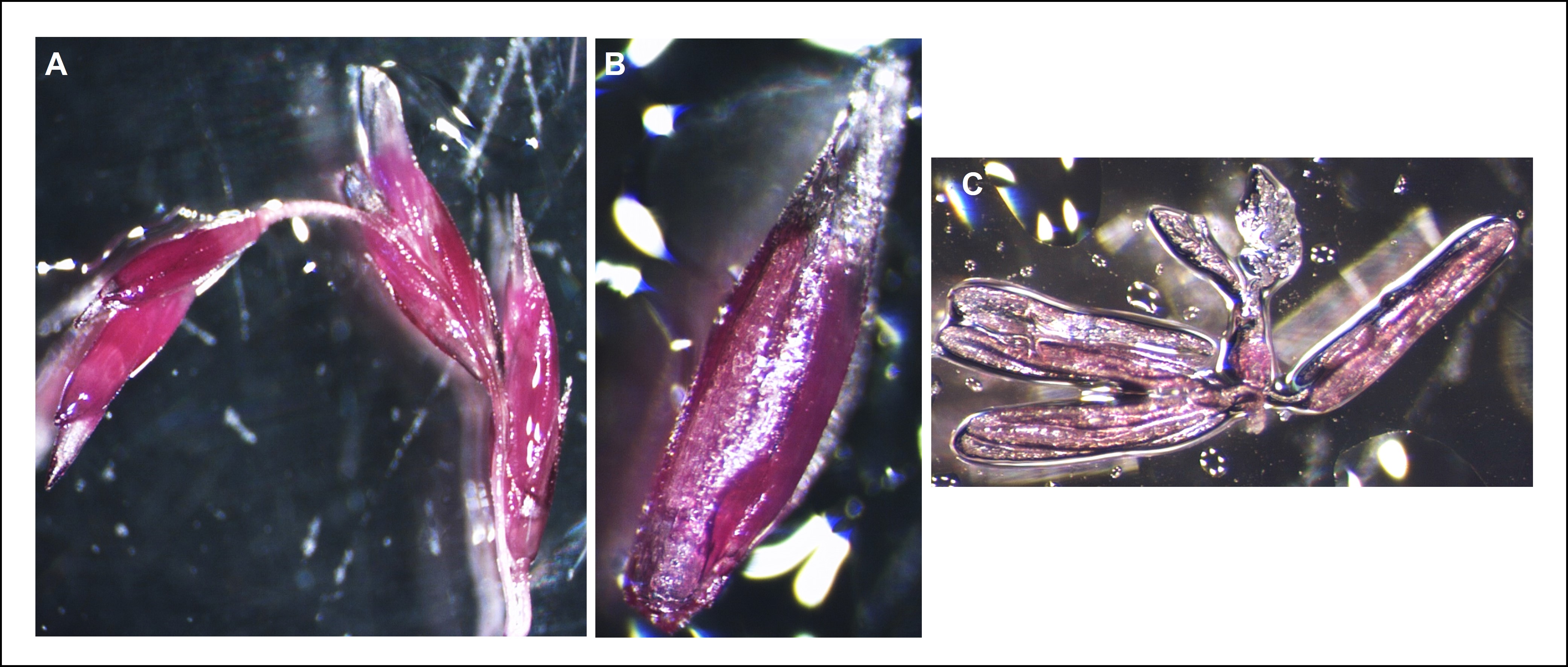

Supplement: Supplementary Figure 1 — Images obtained under the stereomicroscope during preparation of the samples. (A) a rachis containing four stained spikelets; (B) a closed stained spikelet, and (C) an open spikelet, displaying the stained pistil with a feathered stigmas at the center and three anthers on the sides, which was later observed under the confocal laser microscope. [file Image1.jpeg]

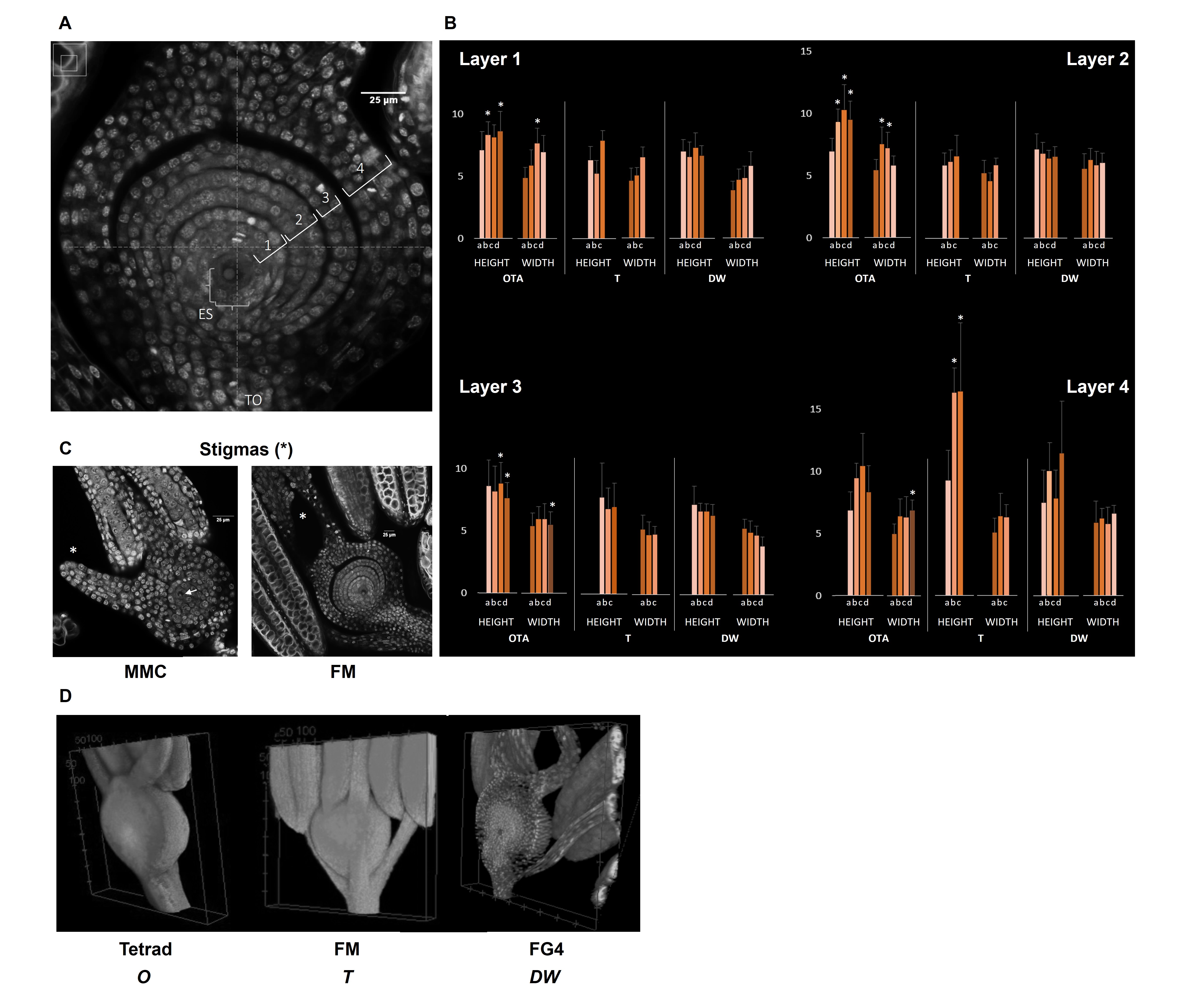

Supplement: Supplementary Figure 2 — (A) References for the measurements of the dimensions of total ovule (TO, dotted lines) and embryo sac (ES, brackets). Four layers analyzed for all stages (parenthesis, 1-4); (B) Bar graphs indicating the comparative measurements of the layer cells (Layer 1-4) between the three contrasting genotypes (OTA, T, DW) for all stages (a, b, c, d): a: MMC; b: FG2, c: FG4, d: FG7. T-test statistical differences between genotypes is shown with a *: p<0.05. (C) Example of the difference in development and size observed in stigmas (indicated with *) of the genotype OTA for the MMC stage (MMC indicated with an arrow), and the FM stage. (D) Constructed 3D confocal models of tetrad stage in sexual OTA (left), FG2 stage in apomictic Tanganyika (middle), and FG4 stage in facultative Don Walter (right). [file Image2.jpeg]

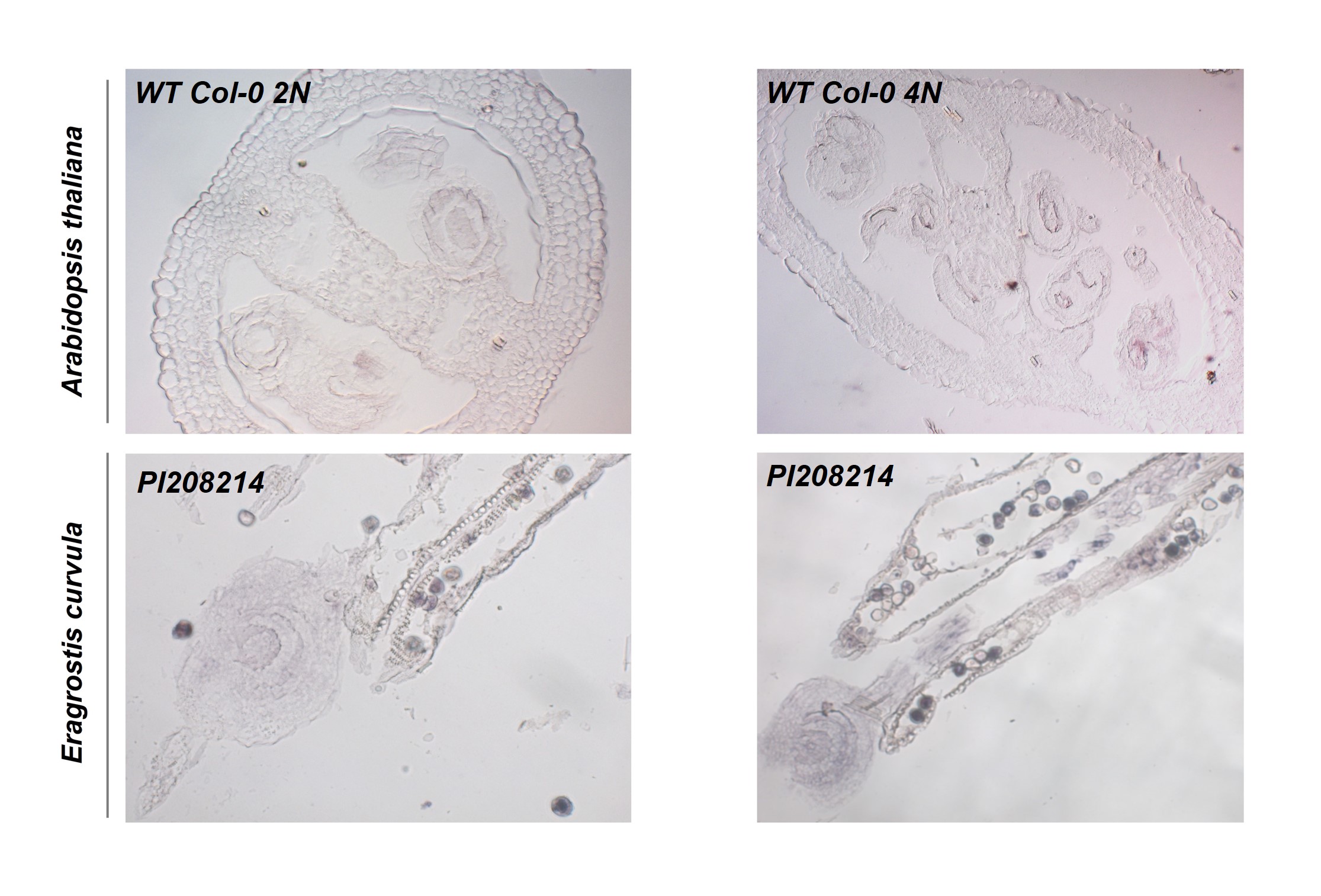

Supplement: Supplementary Figure 3 — Images of the in situ hybridizations with the sense probe performed in A. thaliana, for both diploid and tetraploid backgrounds, and E. curvula sexual diploid genotype. [file Image3.jpg]
